# Machine Learning and Novel Biomarkers for the Diagnosis of Alzheimer’s Disease

**DOI:** 10.3390/ijms22052761

**Published:** 2021-03-09

**Authors:** Chun-Hung Chang, Chieh-Hsin Lin, Hsien-Yuan Lane

**Affiliations:** 1 Institute of Clinical Medical Science, China Medical University, Taichung 40402, Taiwan; chang763@gmail.com; 2 Department of Psychiatry & Brain Disease Research Center, China Medical University Hospital, Taichung 40402, Taiwan; 3 An Nan Hospital, China Medical University, Tainan 709025, Taiwan; 4 Graduate Institute of Biomedical Sciences, China Medical University, Taichung 40402, Taiwan; 5 Kaohsiung Chang Gung Memorial Hospital, Chang Gung University College of Medicine, Kaohsiung 83301, Taiwan; 6 School of Medicine, Chang Gung University, Taoyuan 83301, Taiwan; 7 Department of Psychology, College of Medical and Health Sciences, Asia University, Taichung 41354, Taiwan

**Keywords:** machine learning, deep learning, AI, biomarker, Alzheimer’s disease

## Abstract

Background: Alzheimer’s disease (AD) is a complex and severe neurodegenerative disease that still lacks effective methods of diagnosis. The current diagnostic methods of AD rely on cognitive tests, imaging techniques and cerebrospinal fluid (CSF) levels of amyloid-β1-42 (Aβ42), total tau protein and hyperphosphorylated tau (p-tau). However, the available methods are expensive and relatively invasive. Artificial intelligence techniques like machine learning tools have being increasingly used in precision diagnosis. Methods: We conducted a meta-analysis to investigate the machine learning and novel biomarkers for the diagnosis of AD. Methods: We searched PubMed, the Cochrane Central Register of Controlled Trials, and the Cochrane Database of Systematic Reviews for reviews and trials that investigated the machine learning and novel biomarkers in diagnosis of AD. Results: In additional to Aβ and tau-related biomarkers, biomarkers according to other mechanisms of AD pathology have been investigated. Neuronal injury biomarker includes neurofiliament light (NFL). Biomarkers about synaptic dysfunction and/or loss includes neurogranin, BACE1, synaptotagmin, SNAP-25, GAP-43, synaptophysin. Biomarkers about neuroinflammation includes sTREM2, and YKL-40. Besides, d-glutamate is one of coagonists at the NMDARs. Several machine learning algorithms including support vector machine, logistic regression, random forest, and naïve Bayes) to build an optimal predictive model to distinguish patients with AD from healthy controls. Conclusions: Our results revealed machine learning with novel biomarkers and multiple variables may increase the sensitivity and specificity in diagnosis of AD. Rapid and cost-effective HPLC for biomarkers and machine learning algorithms may assist physicians in diagnosing AD in outpatient clinics.

## 1. Introduction

Alzheimer’s disease (AD), characterized by progressive memory loss and cognitive impairment, is the most common cause of dementia [1]. Approximately 5.7 million American patients suffer from AD. In 2015, AD was ranked as the sixth leading cause of death in the United States. This care is valued at more than $232 billion and contributes as a factor of extended risk of emotional anxiety and negative mental and physical health issues of caregivers. However, early and accurate diagnosis of AD may help save up to $7.9 trillion in medical and care expenses [2].

Current diagnostic approaches for AD mainly depend on neurocognitive tests, brain imaging, and cerebrospinal fluid (CSF) assays [3,4]. Deposition of amyloid plaques, neurofibrillary tangles and significant synapse loss are noted in brain pathology in patients with AD [5]. Diagnostic guidelines have included cerebrospinal fluid (CSF) levels of amyloid-β1-42 (Aβ42), total tau protein and hyperphosphorylated tau (p-tau) [6,7]. CSF biomarkers like Aβ42 and p-tau have been used for research purposes. However, these methods are expensive and relatively invasive [8,9]. Besides, sensitivity and specificity of CSF Aβ42 and p-tau biomarkers have raised concerns about their clinical implication [6,10,11]. The sensitivity of CSF Aβ42 ranges from 0.69 to 0.81 and specificity ranges from 0.44 to 0.89 [12]. Moreover, patients with AD are generally diagnosed late. If AD can be detected in early stages before major brain damage develop, patients may benefit more from treatment. Therefore, identifying biomarkers that can assist in detecting AD early or at onset is critical. Biomarkers can improve diagnostics and enable treatment initiation at the earliest possible stage [13]. 

However, the causal link between Aβ and AD has not been proved. The role of the amyloid-β in the definition, etiology and diagnosis of Alzheimer’s disease is questioned [14]. Pathological levels of Aβ and tau are noted in cognitively normal people. About 20% of cognitively normal elderly exhibit neuropathological AD when restrictive diagnostic criteria for Aβ and tau pathology are applied [15]. On the contrary, some individuals clinically diagnosed with AD do not have Aβ pathology. Studies have reported limited evidence of cerebral AD pathology in approximately 10–20% of individuals clinically diagnosed with AD [16,17].

Therefore, biomarkers according to other mechanisms of AD pathology have been investigated. Neuronal injury biomarker includes neurofiliament light (NFL). Biomarkers about synaptic dysfunction and/or loss includes neurogranin, BACE1, synaptotagmin, SNAP-25, GAP-43, synaptophysin. Biomarkers about neuroinflammation includes sTREM2, and YKL-40 [12]. Besides, abnormal hyperfunction of the N-methyl-d-aspartate receptor (NMDAR) has been found to be involved in synapse dysfunction and neurotoxicity of AD mechanisms [18,19,20]. d-Serine, one of major coagonists at the NMDARs, has been found to be related with NMDAR-mediated neurotoxicity [21,22]. On the other hand, d-serine has been shown to increase adult hippocampal neurogenesis [23]. Several trials have investigated CSF d-serine levels. Two studies have reported that CSF d-serine levels in an AD group were significantly higher than in a control group [24,25].

A machine learning algorithm is one of artificial intellectual techniques for selecting the best model from a set of alternatives to fit a set of observations. Machine learning algorithms have several merits, including nonlinearity, fault tolerance, and real-time operation, thereby making them suitable for complex applications [26]. Machine learning tools are being increasingly used in precision psychiatry [27,28,29]. However, most of current studies have used brain image equipment that is not feasible in clinical practice. A pilot study enrolling 31 healthy controls, 21 patients with MCI, and 133 patients with AD has used machine learning models to predict AD. d-Glutamate is one of agonists at the NMDARs. The naïve Bayes model and random forest model appeared to be the best models for determining MCI and AD susceptibility, respectively (area under the receiver operating characteristic curve: 0.8207 and 0.7900; sensitivity: 0.8438 and 0.6997; and specificity = 0.8158 and 0.9188, respectively). Therefore, in the context of machine learning methods, we review various research studies with novel biomarkers on diagnosis of AD.

## 2. Methods

### Search Strategy

Search Strategy

PubMed, Cochrane Systematic Reviews, and Cochrane Collaboration Central Register of Controlled Clinical Trials databases were searched for studies on machine learning and novel biomarkers for the diagnosis of Alzheimer’s disease from the earliest record to January 2021. Review studies that investigated biomarker for dementia or AD patients were analyzed and included trials and related review articles were manually reviewed for relevant references. The search strings used are the following: “(machine learning OR deep learning OR AI) AND biomarker AND (dementia OR Alzheimer’s disease.” This article reviews and summarizes these clinical trials.

## 3. Results

### 3.1. Machine Learning Models in Alzheimer’s Disease

Machine learning is one of artificial intellectual techniques, used for classification, regression, clustering, or normative modeling. Machine learning algorithms can be divided into supervised models where the data are labeled, unsupervised algorithms where the aim is to separate an unlabeled data into groups of related cases, and semi-supervised algorithms including both labeled and unlabeled data [30]. A machine learning algorithm is a procedure for selecting the best model from a set of alternatives that fit a set of observations. Machine learning algorithms have several merits, including nonlinearity, fault tolerance, and real-time operation, thereby making them suitable for complex applications [26]. 

There are dozens of machine learning algorithms have been developed. Common algorithms are listed in Table 1. In this review, relevant studies with biomarkers and machine learning models are listed in Table 2.

### 3.2. Amyloid-β1-42 (Aβ42), Tau Protein and Hyperphosphorylated Tau (p-tau) with Machine Learning

Several AD biomarkers have been studied including the deposition of pathological amyloid (Aβ) and tau in the cerebrospinal fluid (CSF) [31], the brain metabolic change derived from fluorodeoxyglucose positron emission tomography (FDGPET) [32], and the structural change in the brain morphology measured from the magnetic resonance imaging (MRI) [33]. 

MRI provides a direct measurement of brain structure as possible biomarker to differential a normal control brain and an AD brain. Numerous studies have been conducted to explore the potential of MRI-based AD biomarkers [34]. The recent availability of a large database of individuals with DAT in the Alzheimer’s Disease Neuroimaging Initiative (ADNI) and other openly available databases has improved MRI-based AD biomarkers. Machine learning provide a powerful tool to explore huge brain image data. Pilot studies have tried to use machine learning to quantify structural MRI neurodegeneration patterns of AD into dementia score on 8,834 images from ADNI, AIBL, OASIS, and MIRIAD databases 

Popuri and his colleagues propose a novel biomarker using structural MRI volume-based features. They computed a similarity score for the participant’s structural patterns relative to those observed in the DAT group. They employed ensemble-learning framework combining structural features in most discriminative ROIs to create an aggregate measure of neurodegeneration in the brain. The classifier model was trained on 423 stable normal control (NC) and 330 DAT subjects. In this dataset, clinical diagnosis has the highest certainty. In the next step, they independently validated on 8,834 unseen images from ADNI, AIBL, OASIS, and MIRIAD Alzheimer’s disease (AD) databases. The results revealed promising potential to predict the development of DAT depending on the time-to-conversion (TTC). The prediction of classification on stable versus progressive mild cognitive impairment (MCI) groups achieved an AUC of 0.81 for TTC of 6 months and 0.73 for TTC of up to 7 years. Therefore, their findings may help assessing the presence of AD structural atrophy patterns in normal aging and MCI stages, and monitoring the progression of the patient’s brain along with the disease course.

Abate and his colleagues evaluated a p53-misfolding conformation recognized by the antibody 2D3A8, also named Unfolded p53 (U-p532D3A8+), in 375 plasma samples derived from InveCe.Ab and PharmaCog/E-ADNI longitudinal trials [35]. They used machine learning models with U-p532D3A8+ plasma levels, Mini-Mental State Examination (MMSE) and apolipoprotein E epsilon-4 (APOEε4) to predict AD. Their results showed likelihood risk in InveCe.Ab with an overall 86.67% agreement with clinical diagnosis. These ML models predicted (AUC = 0.92) Aβ+—amnestic Mild Cognitive Impairment (aMCI) patients who will develop AD in PharmaCog/E-ADNI, where subjects were stratified according to Cerebrospinal fluid (CSF) AD markers (Aβ42 and p-Tau). These findings may support U-p532D3A8+ plasma level as a promising additional candidate blood-based biomarker for AD.

### 3.3. PET-Based Tau Biomarker with Machine Learning

Deep learning, developed from machine learning, has been used in a variety of applications in the rapidly growing huge and complicated amount of medical imaging data [36]. Many studies have been focusing on the application of deep learning to AD research. The prediction of AD mainly rely on deep learning using neuroimaging data such as magnetic resonance imaging (MRI) and/or amyloid positron emission tomography (PET). However, MRI scans cannot visualize molecular pathological hallmarks of AD, and amyloid PET cannot, without difficulty, visualize the progression of AD due to the accumulation of amyloid-β early in the disease course with a plateau in later stages [37].

The presence and location of pathological tau deposition in the human brain are well established [38]. Studies have analyzed AD-related neuropathology and generated a staging algorithm to describe the anatomical distribution of tau [39]. Subsequent studies have revealed that the topography of tau corresponds with the pathological stages of neurofibrillary tangle deposition. Cross-sectional autopsy data indicated that AD-related tau pathology begins with tau deposition in the medial temporal lobe (Braak stages I and II), moves to the lateral temporal cortex and part of the medial parietal lobe (stages III and IV), and eventually proceeds to broader neocortical regions (stages V and VI) [40]. Convolutional neural networks (CNNs) are novel and frequently used in deep learning. A CNN combined with tau PET is novel because the resulting spatial characteristics and interpretation differ considerably with those of amyloid PET, FDG PET, or MRI. Furthermore, the regional location and topography of tau PET signals are considered more crucial than those of other molecular imaging modalities, resulting in implications for how a CNN interacts with such complex inputs as well as for the visualization of informative features. 

Choi et al. developed a deep CNN–based automatic image interpretation system that could accurately predict future cognitive decline in patients with MCI by using FDG and florbetapir PET [41]. Their PET images included those of 139 patients with AD, 171 patients with MCI, and 182 healthy controls obtained from the ADNI database. Their deep CNN was trained using three-dimensional (3D) PET volumes of AD and healthy controls as inputs. By contrast, manually defined image feature extraction methods, such as quantification using predefined regions of interest, were unnecessary for our approach. Furthermore, their CNN used minimally processed images without spatial normalization, which is commonly used in conventional quantitative analyses. The cognitive outcomes of patients with MCI were predicted using this CNN. The prediction accuracy of the conversion of MCI to AD was compared with the conventional feature-based quantification approach. Prediction accuracy (84.2%) for conversion to AD in patients with MCI outperformed conventional feature-based quantification approaches. ROC analyses revealed that the performance of the CNN was significantly higher than that of the conventional quantification methods (*p* < 0.05). The output scores of the CNN were strongly correlated with the longitudinal changes in cognitive measurements (*p* < 0.05). These results demonstrate the feasibility of deep learning as a practical tool for identifying predictive neuroimaging biomarkers.

Jo and his colleagues developed a novel deep learning-based framework. They used tau PET to identify the morphological phenotypes of tau deposition [42]. With these tau PET images, they used deep learning models to differentiate patients with AD from healthy controls. This 3D CNN-based classification model yielded an average accuracy of 90.8% according to five-fold cross-validation. In addition, the researchers used a layer-wise relevance propagation (LRP) model to identify the brain regions in tau PET images that contributed the most to the classification results. The most-identified brain regions were the hippocampus, parahippocampus, thalamus, and fusiform. The LRP results were consistent with those from the voxel-wise analysis in SPM12, indicating significant focal AD–associated regional tau deposition in the bilateral temporal lobes, including the entorhinal cortex. The AD probability scores calculated by the classifier were correlated with brain tau deposition in the medial temporal lobe in patients with MCI (r = 0.43 and r = 0.49 for early and late MCI, respectively). A deep learning framework combining 3D CNN and LRP algorithms can be used with tau PET images to identify informative features for AD classification and may be feasible for the early detection of AD during the prodromal stages.

Patients with AD exhibit early changes in the structural integrity of white matter (WM). A pilot study investigated the use of diffusion tensor imaging (DTI) in assessing WM alterations in the predementia stage of mild cognitive impairment (MCI) [43]. They applied a Support Vector Machine (SVM) with DTI and volumetric magnetic resonance imaging data from 35 amyloid-β42 negative MCI subjects (MCI-Aβ42−), 35 positive MCI subjects (MCI-Aβ42+), and 25 healthy controls (HC) retrieved from the European DTI Study on Dementia. The SVM was applied to DTI-derived fractional anisotropy, mean diffusivity (MD), and mode of anisotropy (MO) maps. For comparison, they studied classification based on gray matter (GM) and WM volume. The accuracies were up to 68% for MO and 63% for GM volume when distinguishing between MCI-Aβ42− and MCI-Aβ42+. When separating MCI-Aβ42+ from HC, the accuracy was up to 77% for MD and a significantly lower accuracy of 68% for GM volume. Therefore, their findings suggest that DTI data provide better prediction accuracy than GM volume in predementia AD.

Qiu and his colleagues developed and validated an interpretable deep learning framework for Alzheimer’s disease classification. The model was trained using clinically diagnosed Alzheimer’s disease and cognitively normal subjects from the Alzheimer’s Disease Neuroimaging Initiative (ADNI) dataset (n = 417). They validated on three independent cohorts: the Australian Imaging, Biomarker and Lifestyle Flagship Study of Ageing (AIBL) (n = 382), the Framingham Heart Study (n = 102), and the National Alzheimer’s Coordinating Center (NACC) (n = 582). Their predicting model was consistent across datasets, with mean area under curve values of 0.996, 0.974, 0.876 and 0.954 for the ADNI study, AIBL, Framingham Heart Study and NACC datasets, respectively. Furthermore, their approach exceeded the diagnostic performance of practicing neurologists (n = 11). The high-risk cerebral regions recognized by the model closely with post-mortem histopathological findings. Their framework provides a clinically adaptable strategy for using routinely available imaging techniques such as MRI to generate nuanced neuroimaging signatures for Alzheimer’s disease diagnosis. This approach may be generalized for linking deep learning to pathophysiological processes in human disease [44].

### 3.4. N-Methyl-d-Aspartate Receptor (NMDAR)-Mediated Biomarkers with Machine Learning

N-Methyl-d-aspartate (NMDA) receptors play a very important role in cognitive functions [45]. NMDAR-mediated glutamate is a major excitatory neurotransmitter in the mammalian central nervous system (CNS) [46]. NMDAR and glutamate play fundamental roles in synaptic plasticity and in the underlying molecular mechanisms of learning and memory [47]. Their crucial roles in excitatory neurotransmission indicate that normal signaling disruption through iGluRs is implicated in a wide range of neuropathological disorders and diseases, especially Alzheimer’s disease (AD) [48,49].

l-Amino acids are predominant in Nature. But their molecular chirality, d-amino acids, have been noted to affect protein folding, neuronal proliferation, and brain functional laterality. Recently, d-form amino acids have been found to play a crucial role in cognitive functions and psychiatric disorders [50]. Pilot studies have shown that d-amino acids are novel neurotransmitters [51]. However, studies on d-glutamate are few. Its role in neurocognitive function remains unclear. A pilot human study showed that d-glutamate levels are associated with cognitive functions in patients with AD or MCI [52].

Previous trials have revealed that decreased plasma d-glutamate levels are associated with cognitive impairment in AD [52,53]. A study of 397 participants reported that d-glutamate levels in patients with MCI, and AD were significantly decreased than those of healthy controls (MCI: 1097.8 ± 284.0, mild AD: 1031.9 ± 775.8, moderate to severe AD: 598.3 ± 551.9, healthy elderly: 1620.4 ± 558.2,). Furthermore, they found that MMSE score was significantly correlated with d-glutamate level (adjusted R square = 0.344) [53]. Another trial enrolling 144 patients showed that the d-glutamate level was negatively correlated with the cognitive functions using Alzheimer’s Disease Assessment Scale-Cognitive Subscale (ADAS-cog) behavior scores (r = −0.177, p = 0.034) [52].

These findings raise the next question about the reason why the lower peripheral d-glutamate levels are correlated with cognitive impairment. Brain image studies have explored the relationship between glutamate and cognition. Wong and his colleagues included eight patients with MCI, nine patients with AD, and 16 healthy elderly controls. They found that reduced hippocampal glutamate in MCI and AD was associated with episodic memory performance [54]. Vijayakumari and his colleagues using functional magnetic resonance imaging (fMRI) study to study 15 patients with amnestic MCI and 22 age-, sex-, and education-matched healthy controls. They noted a significant increase was observed in glutamate during a working memory task (both 0 back and 1 back) in healthy controls, but no significant changes were detected in patients with MCI [55].

Pilot studies have used machine learning models with NMDAR-mediated biomarker like d-amino acid oxidase activator (DAOA, also known as G72) protein level to detect schizophrenia. They enrolled 149 participants including 89 patients with schizophrenia and 60 healthy controls. The naive Bayes model using two factors like G72 rs1421292 and G72 protein. The naive Bayes showed the best model for disease susceptibility (sensitivity = 0.7969, specificity = 0.9372, area under the receiver operating characteristic curve (AUC) = 0.9356) [56]. Another machine learning- ensemble approach to predict schizophrenia using biomarker in the *N*-methyl-D-aspartate receptor (NMDAR) and tryptophan catabolic pathways [57]. The analysis revealed that the ensemble boosting model with random under sampling [AUC = 0.9242 ± 0.0652; sensitivity = 0.8580 ± 0.0770; specificity = 0.8594 ± 0.0760] performed best to predict complicated relationship between schizophrenia and biomarkers.

Chang and his colleagues [58] enrolled 31 healthy controls, 21 patients with MCI, and 133 patients with AD. They measured serum d-glutamate levels using high-performance liquid chromatography (HPLC). Cognitive deficit severity was assessed using the Clinical Dementia Rating scale and the MMSE. The researchers employed four machine learning algorithms (SVM, logistic regression, random forest, and naïve Bayes) to build an optimal predictive model for distinguishing patients with MCI or AD from healthy controls. They found that the MCI and AD groups had lower plasma d-glutamate levels (1097.79 ± 283.99 and 785.10 ± 720.06 ng/mL, respectively) than did healthy controls (1620.08 ± 548.80 ng/mL). They found that the naïve Bayes model and random forest model were the best models for determining MCI and AD susceptibility, respectively (area under the receiver operating characteristic curve: 0.8207 and 0.7900; sensitivity: 0.8438 and 0.6997; and specificity = 0.8158 and 0.9188, respectively). Moreover, the total MMSE score was positively correlated with d-glutamate levels (r = 0.368, *p* < 0.001). Multivariate regression analysis indicated that d-glutamate levels were significantly associated with the total MMSE score (B = 0.003, confidence interval: 0.002–0.005, *p* < 0.001). Their results showed that peripheral plasma d-glutamate levels were associated with cognitive impairment and may therefore be a suitable peripheral biomarker for detecting MCI and AD. They suggested rapid and cost-effective HPLC for biomarkers with machine learning models may improve diagnosis of MCI and AD in outpatient clinics.

In addition to predicting Alzheimer’s disease, machine learning models may help to predict NMDAR antagonists for new medication development. Recently, generative deep learning models have been applied to de novo drug design as a means to expand the amount of chemical space that can be explored for potential drug-like compounds. Schultz and his colleagues assessed the application of a generative model to the NMDAR. They investigated two primary objectives. First, the creation and release of a comprehensive library of experimentally validated NMDAR phencyclidine (PCP) site antagonists to assist the drug discovery community. Second, an analysis of both the advantages conferred by applying such generative artificial intelligence models to drug design and the current limitations of the approach. They applied and provided source code for, a variety of ligand- and structure-based assessment techniques used in standard drug discovery analyses to the deep learning- generated compounds. Finally, they found twelve candidate antagonists. Further synthesis and experimental validation of these compounds are still required [59].

### 3.5. Metabolites Biomarkers with Machine Learning

Recently, pilot studies have examined blood metabolites as potential biomarkers for AD [60]. It is easier to evaluate metabolites at blood than at CSF or brain tissue. Blood metabolites may represent an essential aspect of the phenotype of an organism. Therefore, they might act as a molecular fingerprint of AD.

Stamate and hiscolleagues [61] used the data from the European Medical Information Framework for AD Multimodal Biomarker Discovery (EMIF-AD) [62]. They explored eight hundred metabolites using machine learning (ML) algorithms to identify those individuals with AD from dataset. They also compared the effectiveness of blood-based metabolites as a predictor of AD with CSF markers.

In this study, they used ML algorithms including deep learning (DL), extreme gradient boosting (XGBoost), and random forest (RF) algorithm. They enrolled 242 cognitively normal (CN) participants and 115 patients with AD-type dementia. Three machine learning models like DL, XGBoost, and RF were used to differentiate AD from CN. They internally validated these models using nested cross validation (NCV). When using the test data, DL showed the AUC of 0.85 (0.80 to 0.89), XGBoost showed 0.88 (0.86 to 0.89) and RF showed 0.85 (0.83 to 0.87). When using CSF amyloid, p-tau and t-tau (together with age and gender), XGBoost revealed the AUC values of 0.78, 0.83 and 0.87, respectively.

**Table 2:** Relevant studies on the biomarkers and predictive models of AD.

**Study**	**Biomarker**	**Model**	**Results**
Popuri et al., 2020 [63]	CSF [t-tau/Aβ1-42]	ensemble-learning	Classification performance on stable versus progressive mild cognitive impairment (MCI) groups achieved an AUC of 0.81 for TTC of 6 months and 0.73 for TTC of up to 7 years, achieving state-of-the-art results.
Abate et al., 2020 [35]	p53	Regression Tree (RT)	These algorithms also accurately classify (AUC = 0.92) Aβ+—amnestic Mild Cognitive Impairment (aMCI) patients who will develop AD
Choi et al., 2018 [41]	amyloid	convolutional neural network (CNN)	Accuracy of prediction (84.2%) for conversion to AD in MCI patients outperformed conventional feature-based quantification approaches.
Jo et al., 2020 [42]	tau	convolutional neural network (CNN)	Deep learning-based classification model of AD from CN yielded an average accuracy of 90.8%
Dyrba et al., 2015 [43]	amyloid-β42	Support Vector Machine (SVM)	accuracies of up to 68% for MO and 63% for GM volume when it came to distinguishing between MCI-Aβ42− and MCI-Aβ42+.
Chang et al. (2021) [1]	D-glutamate	support vector machine, logistic regression, random forest, and naïve Bayes	The naïve Bayes model and random forest model appeared to be the best models for determining MCI and AD susceptibility, respectively (area under the receiver operating characteristic curve: 0.8207 and 0.7900; sensitivity: 0.8438 and 0.6997; and specificity = 0.8158 and 0.9188, respectively).
Stamate et al. 2019 [61]	Metabolites biomarkers	Deep Learning (DL), Extreme Gradient Boosting (XGBoost) and Random Forest (RF)	DL produced the AUC of 0.85 (0.80–0.89), XGBoost produced 0.88 (0.86–0.89) and RF produced 0.85 (0.83–0.87).

## 4. Outlook and Future Direction

AD is human diseases. In addition to biomarkers in brain, CSF or blood, multiple risk factors with several markers are under study. In addition to medications, multidomain interventions, targeting multiple risk factors simultaneously, could be effective dementia prevention strategies. However, multidomain interventions may be burdensome and not universally acceptable. Coley and his colleague [64] investigated adherence rates and predictors for all intervention components separately and simultaneously in the Finnish Geriatric Intervention Study to Prevent Cognitive Impairment and Disability (FINGER) and Multidomain Alzheimer Preventive Trial (MAPT). FINGER participants received a 2-year multidomain lifestyle intervention (physical training, cognitive training, nutritional counseling, and cardiovascular monitoring). MAPT participants received a 3-year multidomain lifestyle intervention (cognitive training, physical activity counseling, and nutritional counseling) with either an omega-3 supplement or placebo. Adherence decreased with increasing intervention complexity and intensity. It was highest for cardiovascular monitoring, nutritional counseling, and the omega-3 supplement, and lowest for unsupervised computer-based cognitive training. The most consistent baseline predictors of adherence were smoking and depressive symptoms. Reducing participant burden, maintaining in-person contacts, and taking into account participant characteristics may increase adherence in future trials with large sample size.

Increasing studies are investigating machine learning techniques with novel biomarkers as promising approaches to predict AD. Some studies have shown promising results. However, some lack large sample sizes and the appropriate power, or not hypothesis-driven. This review examined novel biomarkers including amyloid, tau protein, NMDAR-mediated biomarkers, and metabolites biomarkers. Because many machine learning models have no standard settings and guidelines, a robust comparison of these trials remains incomplete. However, brain image-based biomarkers with machine learning models especially deep learning such as CNN are promising. Moreover, machine learning combined with NMDAR-mediated biomarkers appear to be a new approach to predict the long-term cognitive outcome. Therefore, although the field of machine learning is relatively immature, such techniques, especially deep learning, warrant further study for their diagnostic and therapeutic implications on patients with AD.

## Figures and Tables

**Table 1:** Common machine learning algorithms.

**Algorithm**	**Learning Type**	**Class**	**Restriction Bias**	**Preference Bias**
**K-Nearest** **Neighbors**	Supervised	Instance based	Generally suitable for measuring distance-based approximations; however, it is subject to dimensionality	Preferred for distance-based problems
**Naive Bayes**	Supervised	Probabilistic	Works on problems where the inputs are independent from each other	Preferred for problems in which the probability is always greater than zero for each class
**Decision Trees/** **Random Forests**	Supervised	Tree	Becomes less useful on problems with low covariance	Preferred for problems with categorical data
**Support Vector** **Machines**	Supervised	Decision boundary	Works where there is a definite distinction between two classification	Preferred for binary classification problems
**Neural Networks**	Supervised	Nonlinear functional approximation	Little restriction bias	Preferred for binary inputs
**Hidden Markov** **Models**	Supervised/ Unsupervised	Markovian	Generally works well for system information where the Markov assumption holds	Preferred for time-series data and memoryless information
**Clustering**	Unsupervised	Clustering	No restriction	Preferred for data that is in groupings given some form of istance (Euclidean, Manhattan, or others)
**Feature Selection**	Unsupervised	Matrix factorization	No restriction	Depending on algorithm can prefer data with high mutual information
**Feature** **Transformation**	Unsupervised	Matrix factorization	Must be a nondegenerate matrix	Will work much better on matrices that don’t have inversion issues
**Bagging**	Meta-heuristic	Meta-heuristic	Will work on just about anything	Preferred for data that is not highly variable
